# S42. KETAMINE DYSREGULATES TASK-RELATED NEURAL OSCILLATIONS IN THALAMO-CORTICAL CIRCUITS: IMPLICATIONS FOR PATHOPHYSIOLOGICAL THEORIES OF VISUAL-PERCEPTUAL DEFICITS IN SCHIZOPHRENIA

**DOI:** 10.1093/schbul/sby018.829

**Published:** 2018-04-01

**Authors:** Tineke Grent-’t-Jong, Davide Rivolta, Joachim Gross, Ruchika Gajwani, Stephen Lawrie, Matthias Schwannauer, Tonio Heidegger, Michael Wibral, Wolf Singer, Andreas Sauer, Bertram Scheller, Peter Uhlhaas

**Affiliations:** 1 University of Glasgow, Institute of Neuroscience and Psychology; 2 School of Psychology, University of East London; 3 Institute of Health and Wellbeing, University of Glasgow; 4 University of Edinburgh; 5 Goethe University; 6 Max-Planck Institute for Brain Research

## Abstract

**Background:**

Hypofunction of the N-methyl-D-aspartate receptor (NMDA-R) has been implicated as a possible mechanism underlying cognitive deficits and aberrant neuronal dynamics in schizophrenia (ScZ).

**Methods:**

In a single-blind cross-over design, 14 participants received either a subanaesthetic dose of S-ketamine (0.006 mg/Kg) or saline while Magnetoencephalographic (MEG) data were recorded during a visual task. In addition, MEG-data were obtained in a sample of unmedicated first-episode psychosis (FEP) patients (n = 10) and in patients with chronic ScZ (n = 16). MEG-data were analyzed at source-level in the 1–90 Hz frequency range in occipital and thalamic regions of interest (ROIs). In addition, directed functional connectivity analysis was performed using Granger Causality (GC). Psychopathology was assessed by the Positive and Negative Syndrome Scale (PANSS).

**Results:**

Behavioral impairments were accompanied by increased amplitude and frequency of gamma-power (63–80 Hz) in occipital regions during Ketamine administration, while low-frequency (~5–30 Hz) activity was upregulated. Moreover, Ketamine disrupted feedforward (FF) and feedback (FB) signaling at high and low frequencies leading to hypoconnectivity in thalamo-cortical (TC) networks. In contrast, FEP and chronic ScZ patients showed a different pattern of MEG-activity, characterized by decreased task-induced high-gamma band oscillations and increased FF/FB-mediated GC-connectivity.

**Discussion:**

The effects of Ketamine on high-frequency oscillations and their connectivity profile are not consistent with observations in FEP and chronic ScZ-patients. Accordingly, the current data have implications for theories of cognitive dysfunctions and circuit impairments in the disorder, suggesting that the effects of acute NMDA-R hypofunction are not consistent with impairments in visuo-perceptual oscillations in ScZ-patients.

